# Celiac Disease Is a Rare Cause of Benign Duodenal Stricture: A Case Report

**DOI:** 10.7759/cureus.37613

**Published:** 2023-04-15

**Authors:** Ibrahim Balubaid

**Affiliations:** 1 Department of Medicine, Division of Gastroenterology, Western University, London, CAN; 2 Department of Medicine, Division of Gastroenterology, King Abdulaziz University Faculty of Medicine, Jeddah, SAU

**Keywords:** peptic ulcer disease, endoscopy, duodenal ulceration, benign duodenal stricture, celiac disease

## Abstract

Duodenal stricture is a rare manifestation of celiac disease. In this case report, we present a case of a 64-year-old male with a history of duodenal stricture proven on both endoscopy and imaging, initially not responsive to endoscopic dilation. Further investigation and biopsy confirmed the diagnosis of celiac disease. In addition to endoscopic treatment, a gluten-free diet resulted in clinical, endoscopic, and histologic improvement. This case highlights the importance of considering celiac disease in the differential diagnosis for patients with duodenal strictures.

## Introduction

Benign duodenal strictures are an uncommon problem encountered by gastroenterologists. Peptic ulcer disease (PUD) is a significant cause of benign duodenal stricture, which is often caused by Helicobacter pylori (H. pylori) and the use of non-steroidal anti-inflammatory drugs (NSAIDs) [1-4]. Duodenal strictures have also been reported in other benign conditions such as inflammatory bowel disease (IBD), pancreatitis, and congenital malformation [5-7]. Precise estimates on the incidence of benign duodenal strictures are lacking. However, it is likely to have decreased recently with the diagnosis and eradication of H. pylori, early diagnosis of PUD, and using proton pump inhibitors (PPIs) to treat upper gastrointestinal inflammation. Common clinical symptoms of duodenal strictures include early satiety, nausea, vomiting, and weight loss [8]. We present a case of a patient with a refractory web-like stricture in the second part of the duodenum (D2) caused by celiac disease.

This article was previously presented as a scientific poster at the Canadian Digestive Disease Week (CDDW) on February 29, 2020.

## Case presentation

**Figure 1:**
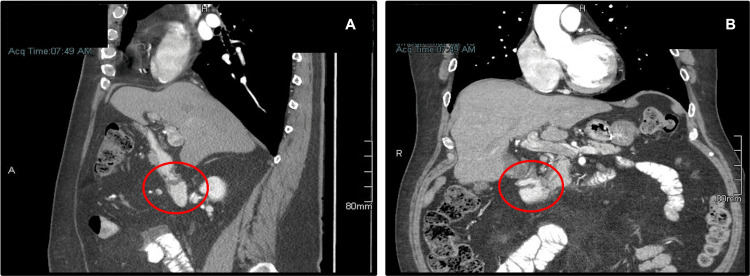
CT scan of the abdomen showing stricture at the level of the proximal second part of the duodenum described as a “duodenal band” A: sagittal view; B: coronal view

A 64-year-old male was referred for consideration of duodenal stenting of a benign refractory stricture in the second part of the duodenum. His medical history includes coronary artery disease, hypertension, dyslipidemia, and previous laparoscopic cholecystectomy. There was no prior history of gastrointestinal disorders. He had a one-year history of abdominal pain, early satiety, and weight loss (10 pounds). He also reported intermittent episodes of diarrhea. His physical exam at the time of the referral was unremarkable, except for evidence of surgical scars from a previous laparoscopic cholecystectomy. Initial laboratory investigations showed mild normocytic anemia (hemoglobin 126. g/L), normal white blood cell count, mean corpuscular volume, and thrombocyte count. His serum electrolytes, transaminases, lipase, urea nitrogen, and creatinine levels were normal. Ferritin was 20 ug/L, lower than the normal limit (normal value 30-300 ug/L). Testing for H. pylori was negative, and he denied NSAIDs use. He received a computed tomography scan (CT scan) of the abdomen, which showed a stricture at the level of the proximal second part of the duodenum described as a “duodenal band” (Figure 1).

**Figure 2:**
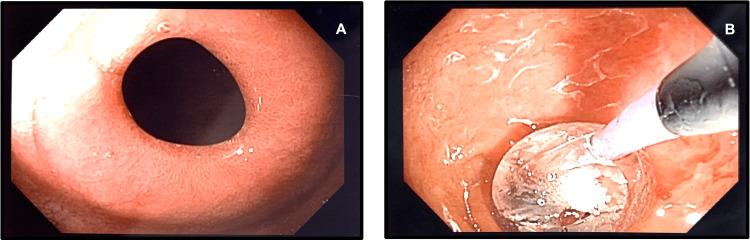
Upper endoscopy evaluation proximal to the duodenal stricture before the endoscopic dilation (A) Upper endoscopy showing a tight web-like stricture in the proximal second part of the duodenum; (B) Endoscopic balloon dilation of the web-like stricture in the second part of the duodenum

Before his referral, two previous attempts of endoscopic balloon dilation were performed with a 12 mm to 16.5 mm balloon and had not resulted in sustained symptomatic or endoscopic improvement. Upper endoscopy was performed to assess the stricture before considering endoscopic stenting as requested. Upper endoscopy showed a tight web-like stricture in the proximal second part of the duodenum (Figure 2). The stricture was balloon dilated again up to 16.5 mm, enabling the endoscope to pass beyond it (Figure 2).

**Figure 3:**
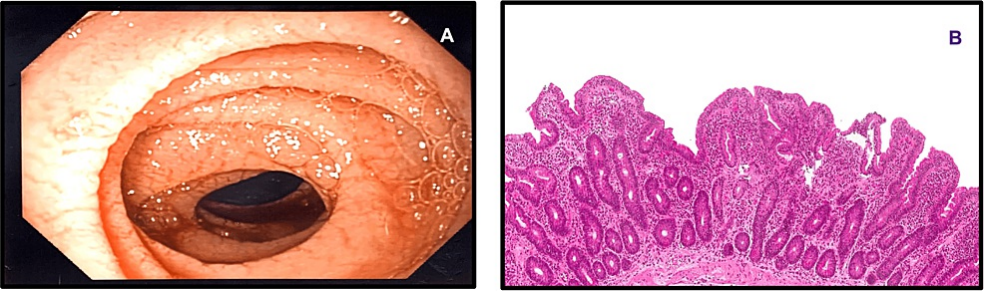
Upper endoscopy and duodenal biopsies of the second part of the duodenum before starting on a gluten-free diet (A) Upper endoscopy showing atrophic mucosa with evidence of loss of villi and scalloping of the folds distal to the stricture; (B) Biopsies from the second part of the duodenum revealing moderate villous blunting and intraepithelial lymphocytosis (Marsh 3a)

The mucosa in the second part of the duodenum distal to the stricture was atrophic, with evidence of loss of villi and scalloping of the folds. Biopsies from D2 distal to the stricture revealed moderate villous blunting and intraepithelial lymphocytosis (Marsh 3a) (Figure 3)

**Figure 4:**
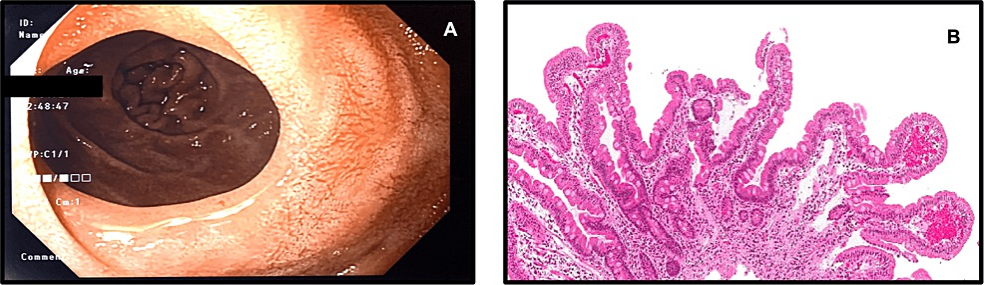
Follow-up upper endoscopy and duodenal biopsies after treatment (A) Endoscopic improvement at the stricture in the second part of the duodenum; (B) Normalization of villous structure (Marsh 0)

Celiac serology testing was abnormal, with an anti-tissue transglutaminase antibody (anti-tTG Ab) level of 32 RU/ml, confirming the diagnosis of celiac disease. Once the diagnosis was confirmed, he was placed on a gluten-free diet, and both endoscopic balloon dilation and a gluten-free diet resolved his symptoms. Initial follow-up endoscopy at a four-month interval revealed improvement in the stricture and normalization of his duodenal folds and duodenal biopsies (Marsh 0) (Figure 4). The anti-tTG Ab level also normalized. The stricture improved long-term, as he continued to be on a gluten-free diet.

## Discussion

This case describes a very uncommon complication of celiac disease. It also highlights the importance of recognizing the endoscopic appearance of celiac disease in patients undergoing endoscopy for other reasons. H. pylori is a major cause of duodenal ulceration and stricture [1]. Other possibilities include NSAIDs or other acid hypersecretory states like Zollinger-Ellison syndrome [9,10]. Our patient tested negative for H. pylori and has no history of NSAID use nor a previous diagnosis of hypersecretory conditions. The likely pathophysiology, in this case, includes gluten-induced mucosal damage causing mucosal inflammation and ulceration in patients with celiac disease, resulting in a benign stricture [11].

A few case reports described benign duodenal stricture as a late complication in advanced cases of celiac disease [11,12]. One case report described an atypical case of celiac disease, which remained in remission on a strict gluten-free diet for six years before developing complications of intestinal ulcerations and stricture required to be managed surgically [12]. Other case reports have reported duodenal stricture preceding the diagnosis of celiac disease. For example, a case series described an association between duodenal stenosis and celiac disease in five patients. In three of these cases, the diagnosis of celiac disease was not expected, and the finding of duodenal stricture preceded the diagnosis of celiac disease [13]. Another case reported a duodenal ulcerative lesion and stricture in young women before confirming the diagnosis of celiac disease [14]. Despite her atypical symptoms, these findings were resolved completely after being on a gluten-free diet.

It is important to note that duodenal strictures are rare in people with celiac disease, and most do not develop strictures. However, it is crucial to be aware of the potential complication. In this case, celiac disease is most likely the cause of duodenal stricture, especially in the absence of PUD from H. pylori or NSAID use. Additionally, the combination of a gluten-free diet and balloon dilation was more effective at improving the endoscopic appearance and symptoms than balloon dilation alone, which had been tried before his diagnosis of celiac disease. Finally, severe refractory strictures in celiac disease have been managed surgically in a few reported cases [12,13].

## Conclusions

In conclusion, a duodenal stricture is a rare manifestation of celiac disease. It can present as a late complication and, in some cases, may even precede the diagnosis of celiac disease. Thus, obtaining duodenal biopsies along with celiac disease serology should be considered in patients with duodenal strictures.
